# Supplementary material for: An Inflammatory Signature to Predict the Clinical Benefit of First-Line Cetuximab Plus Platinum-Based Chemotherapy in Recurrent/Metastatic Head and Neck Cancer
Source: Cells. 2022 Oct 10;11(19):3176. doi: 10.3390/cells11193176 (PMC9563947; doi:10.3390/cells11193176)
Supplement: Supplementary file 1 [file cells-11-03176-s001.zip › cells-1925637-supplementary.pdf]

Supplementary Table S1		Univariable analysis		Multivariable analysis	
**PFS**		**HR (95% CI)**	**P-value**	**HR (95% CI)**	**P-value**
**inflammatory signature**	high score group (reference)		0.0025		0.00069
**inflammatory signature**	low score group	2.35 (2.10-2.59)		2.4 (1.49-3.9)	
**inflammatory signature**	intermediate score group	1.42 (1.19-1.66)		1.5 (0.92-2.4)	
**ECOG PS**	1 vs 0	1.28 (1.08-1.48)	0.213	1.5 (0.98-2.2)	0.062
**subsite**	oropharynx vs other	1.52 (1.33-1.72)	0.031	1.5 (1.04-2.3)	0.031

		Univariable analysis		Multivariable analysis	
**OS**		**HR (95% CI)**	**P-value**	**HR (95% CI)**	**P-value**
**inflammatory signature**	high score group (reference)		0.00022		0.00011
**inflammatory signature**	low score group	3 (2.73-3.27)		3.3 (1.92-5.7)	
**inflammatory signature**	intermediate score group	1.37 (1.1-1.63)		1.5 (0.89-2.5)	
**ECOG PS**	1 vs 0	1.39 (1.18-1.6)	0.117	1.7 (1.08-2.5)	0.02
**subsite**	oropharynx vs other	1.31 (1.1-1.52)	0.193	1.3 (0.88-2.0)	0.178
